# Exploration on Scientific Research Data-Targeted Intelligent Recommendation System Using Machine Learning Under the Background of Sustainable Development

**DOI:** 10.3389/fpsyg.2022.788183

**Published:** 2022-02-16

**Authors:** Ruoqi Wang, Shaozhong Zhang, Lin Qi, Jingfeng Huang

**Affiliations:** ^1^School of Information and Intelligent Engineering, Zhejiang Wanli University, Ningbo, China; ^2^Department of Scientific Research, Zhejiang Wanli University, Ningbo, China

**Keywords:** education scientific research data, content-based recommendation, intelligent recommendation system, collaborative filtering, sustainable development

## Abstract

The purpose is to provide researchers with reliable Scientific Research Data (SRD) from the massive amounts of research data to establish a sustainable Scientific Research (SR) environment. Specifically, the present work proposes establishing an Intelligent Recommendation System (IRS) based on Machine Learning (ML) algorithm and SRD. Firstly, the IRS is established over ML technology. Then, based on user Psychology and Collaborative Filtering (CF) recommendation algorithm, a hybrid algorithm [namely, Content-Based Recommendation-Collaborative Filtering (CBR-CF)] is established to improve the utilization efficiency of SRD and Sustainable Development (SD) of SR. Consequently, the present work designs literature and SRD-targeted IRS using the hybrid recommendation under the background of SD. The proposed system’s feasibility is analyzed through experiments. Additionally, the system performance is analyzed and verified from accuracy, diversity, coverage, novelty, and recommendation efficiency. The results show that the hybrid algorithm can make up for the shortcomings of a single algorithm and improve the recommendation efficiency. Experiments show that the accuracy of the proposed CBR-CF algorithm is the highest. In particular, the recommendation accuracy for the single-user system can reach 82–93%, and the recall of all recommended algorithms falls between 60 and 91%. The recall of the hybrid algorithm is higher than that of a single algorithm, and the highest recall is 91%. Meanwhile, the hybrid algorithm has comprehensive coverage, good applicability, and diversity. Therefore, SD-oriented SRD-targeted IRS is of great significance to improve the SRD utilization and the accuracy of IRS, and expand the achievement value of SR. The research content provides a reference for establishing a sustainable SR environment and improving SR efficiency.

## Introduction

With the continuous deepeninga of Scientific Research (SR), the Data Volumes (DV) accumulate over time, causing trouble for researchers to excavate adequate information from Scientific Research Data (SRD). On the other hand, ecologists’ research on the future development of the Natural Environment (NE) has given birth to the concept of Sustainable Development (SD). Specifically, SD refers to planned development to meet contemporary people’s living and production needs without impacting future generations’ wellbeing. Therefore, the concept of SD can be used to balance the relationship between economic development and natural resources and between human production and NE (Yin et al., 2014). Today, the concept of SD has seen wide applications in various fields. Experts from all social spheres also begin to define SD from the social attribute, economic attribute, Science and Technology (S&T) attribute, and so on. In other words, researchers try to establish a sustainable social ecology based on the coordination and joint development of society, economy, population, resources, and environment (Lélé, 1991).

Therefore, the research on SD needs to learn from the academic research achievements in multiple disciplines. The purpose is to solve the problems that, given huge DV, users cannot mine the required information from the massive amounts of SRD in time. The present work employs computer algorithms to improve the efficiency of researchers in finding relevant data, improve the efficiency of people’s Decision-Making (DM), and further promote the progress of SR (Zheng et al., 2018). Meanwhile, information dispersibility and heterogeneity of relevant research will be highly intertwined and integrated with further social development (Muhaisen et al., 2019).

Applying the SD idea to the scientific field can expand the information Utilization Rate (UR) and promote sustainable S&T development. To this end, the present work studies the Intelligent Recommendation System (IRS) based on SRD to help people retrieve the required information quickly and accurately (Chen, 2019). At present, personalized recommendation technology has become an effective method to deal with information overload, including Content-Based Recommendation (CBR), Collaborative Filtering (CF) recommendation, hybrid recommendation, knowledge-based recommendation, network structure-based recommendation, and social-tag based recommendation (Bruin and Florida, 2017). However, the rapidly developing Electric Commerce (e-commerce) puts higher requirements for user recommendation that cannot be achieved through a single recommendation technology. Accordingly, some scholars propose to mix various recommendation technologies to make up for the shortcomings of a single algorithm to improve the information recommendation quality. In particular, some attempt to combine CF technology with other technologies for user recommendation services (Chaudhary and Roy Chowdhury, 2019). Given the scholars’ user Psychology in pursuit of highly relevant SR information, the present work will design the SRD-targeted IRS through the optimization algorithm combined with the user’s psychological model to pursue personalized recommendation.

Therefore, to establish an SRD-targeted IRS under the background of SD, based on the existing research results, the present work designs the SRD-targeted IRS from the aspects of the principle, performance, architecture design, algorithm selection, and system module design. Then, Machine Learning (ML) technology is introduced to improve the IRS’s accuracy. Significantly, the present work combines ML technology with user Psychology to create a targeted user curiosity psychological model to effectively recommend personalized information for users. The designed IRS can improve the information mining efficiency for SRD and promote the SD of SR. Therefore, the present work constructs an SD-oriented IRS using SRD, improving the accuracy and efficiency of recommendation and providing analysis tools for various fields.

In actual applications, the designed IRS can provide users with corresponding SRD or literature according to the imported user’s Point Of Interest (POI) and reduce the data collection time. The innovation is to apply ML technology to the construction of SRD-targeted IRS and combines user Psychology to improve the recommendation accuracy. The research content has made a certain contribution to the construction of IRS for researchers’ SRD and improving the quality and efficiency of information recommendation under the background of SD.

The present work will be carried out in four parts. Firstly, it analyzes and explains the research status in related fields and leads to the methods to solve the existing problems. Secondly, it examines the research theories related to ML and puts forward the research methods. Then, combined with the appropriate research methods, the algorithm is designed, trained, and tested to verify the effectiveness of the designed algorithm. Finally, combined with the experimental results, the designed algorithm and IRS are analyzed and summarized to prove its effectiveness in addressing the related problems.

## Literature Review

Under the research background of SD, how to effectively recommend SRD and establish efficient recommendation methods is very important in the current academia. Currently, although the information sharing efficiency is low, many scholars still share their SRD as much as possible to achieve more research works. Colavizza et al. (2020) reasoned that many journals encouraged authorized authors to provide descriptions of data availability in the literature. Then, according to the data of more than 500,000 journal articles published by PLOS and BMC, they established an automatic labeling system of Data Availability Statement (DAS) based on four categories. The regression analysis could analyze the citation advantages of different sentence categories. After the release of the mandatory policy, the DAS had become common. Then, more than 88% of the articles were published with DAS in 2018. These data showed promising results in the reference prediction model, providing data support for follow-up research. On the other hand, data play an essential role in qualitative analysis. For example, scholars in medical circles use historical data for comparative analysis. Roberts et al. (2019) argued that the preciseness of qualitative data analysis should be determined through detailed interpretation. For researchers and students to use the data of previous master’s and doctoral theses, qualitative and quantitative methods should be used for data connection analysis to ensure data reliability.

With the continuous development of Big Data Analytics (BDA) technology, IRS has also become the focus of many fields. Lin et al. (2019) suggested that the development of Information Technology (IT) had promoted the application of the intelligent classroom model. At the same time, it also improved the college students’ comprehensive quality and ability. Considering the complexity of teaching mode and students’ personalities, the intelligent classroom model based on adaptive learning resource recommendation helped improve students’ learning efficiency and provide a reference for the teaching quality of professional courses in higher institutions. Perumal et al. (2019) proposed a new recommendation system that refined the final frequent item patterns that evolved from Frequent Pattern Mining (FPM) technology. Then, fuzzy logic divided the final content into three levels to provide appropriate content. Compared with traditional methods, the proposed method had higher efficiency and accuracy. Shahbazi et al. (2020) studied an algorithm based on CF to process the users’ big data from symmetrical purchase orders and repeated purchase behaviors. The algorithm improved the accuracy of predicting the related products recommended to potential customers.

Content-based recommendation is a more effective recommendation method to date, so it sees wide applications in various research fields. Leśniak and Zima (2018) believed that more construction projects adopted the SD concept, which affected the cost of construction projects. Therefore, they proposed a method of estimating the construction cost of sports venues using case-based reasoning. The error of this method was 14%, which provided good results for the preliminary calculation. The results were of great significance to reduce the building’s construction cost. Wang et al. (2020) studied a hybrid recommendation algorithm integrating user and project content information and matrix decomposition. The algorithm could predict and evaluate the deviation of users or projects with the content information of users and projects. Cai et al. (2020) analyzed a multi-objective hybrid recommendation model based on the score to simultaneously optimize the accuracy, recall, diversity, novelty, and coverage of recommendation and improve the multi-objective evolutionary algorithm and the model performance based on the partition-based knowledge mining strategy.

To sum up, SRD often sees applications in the medical field or data feasibility analysis. Recommendation systems use open-source data, but SRD is rarely used to establish recommendation models. Moreover, scholars in various research fields have researched management methods for either SRD recommendation or user content recommendation separately. However, few studies are combining the two, and the recommended content also needs users’ further confirmation, which affects the efficiency of SR. The designed recommendation algorithm can help reduce the time for scholars to collect SRD and realize the recyclable data collection and output process.

Based on the above analysis, the present work mainly uses SRD to explore the construction of IRS under the background of SD to obtain a recommendation system with higher accuracy and efficiency. Specifically, there is a need to design and analyze the personalized recommendation for SRD under the background of SD. Accordingly, the SRD-oriented personalized recommendation is carried out based on ML technology and user Psychology, thereby reducing the time of information retrieving while improving the efficiency of information mining for SRD and promoting the SD of SR.

## Materials and Methods

### Machine Learning-Based Intelligent Recommendation System

Personalized recommendation systems mainly involve User Behavior Analytics (BA), users’ POI excavation, and information recommendation. Standard personalized recommendation algorithms include the CF recommendation algorithm, CBR algorithm, Association Rules (AR) recommendation algorithm, and hybrid recommendation algorithms, such as Content-Based Recommendation-Collaborative Filtering (CBR-CF). The present work selects the ML algorithm to design IRS. Ml is an important research field of Artificial Intelligence (AI). In particular, ML algorithm can effectively simulate the human brain’s Neural Network (NN) for analysis and learning. It can also mimic the mechanism of the human brain to realize data interpretation. For example, the Deep Neural Network (DNN) can extract and express the data features.

In essence, ML is a learning process that simulates the way of thinking of the human brain, which features the development process from shallow understanding to deep understanding. Ml has an outstanding learning ability and can effectively mine the original data’s feature attributes or feature expression. ML model can achieve good results in large-scale training data, including Deep Belief Network (DBN), deep Boltzmann Machine (BM), Stacked Auto Encoder (SAE), and Convolutional Neural Network (CNN). A personalized learning resource recommendation system can effectively improve the learning effect, realize customized learning, and fully reflect the learner-centered philosophy.

The ML-based personalized learning resource recommendation system can accurately recommend learning resources according to users’ needs, hobbies, and POI to help learners learn accurately (Guo et al., 2017). A single recommendation algorithm also has unique advantages and disadvantages. To name a few, Case-based Reasoning (CBR) does not depend on the user’s score and can recommend related content according to users’ POI, which is not affected by the data sparsity. However, the CBR algorithm is limited by text format and cannot be recommended across types (Gavalas et al., 2014). CF algorithm can obtain implicit user POI, be used for complex text types, and have extensive promotion and novel recommendation information.

**FIGURE 1:**
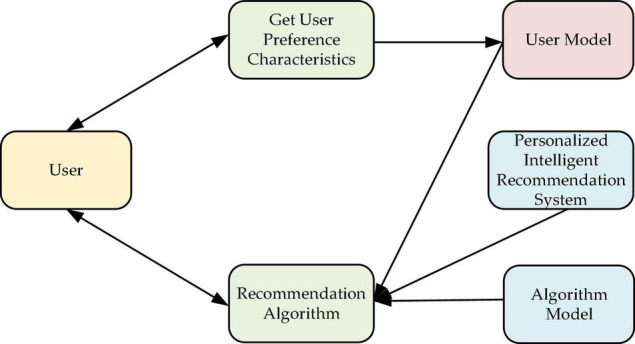
Personalized IRS model.

Yet, the CF algorithm is limited by the cold-start problem, data sparsity, and system scalability. Therefore, combining CBR and CF (the CBR-CF hybrid recommendation technology) can solve the problems of data sparsity and the cold start of a single recommendation system and expand the range of text types. According to relevant literature (Victor, 2009), any hybrid algorithm can significantly improve coverage and automation, improve timeliness and operation efficiency, and preliminarily realize the “intelligent” design of the system. Because users’ research directions and demands for SRD differ significantly, independent recommendation modules can be selected to combine design algorithm with ML technology and user Psychology. The independent recommendation module can provide different recommendation schemes for different situations, meet the diversified user needs, and improve the system processing efficiency. Figure 1 draws the basic framework of personalized IRS design.

As shown in Figure 1, the user is both the output point of the model data and the recipient of the design algorithm. The designed model can realize a positive feedback process by analyzing the user’s data and using the designed algorithm model to provide personalized recommendation services to users.

The designed hybrid recommendation system comprises the early stage and late stage. Of these, the early stage mainly collects the information of User Behavior (UB) characteristics and predicts them. Comparatively, data collection and prediction in the early stage take a long time. Later, the recommendation algorithm calculates the data and recommends the related content to users. The hybrid recommendation system divides users into historical users and new users.

**FIGURE 2:**
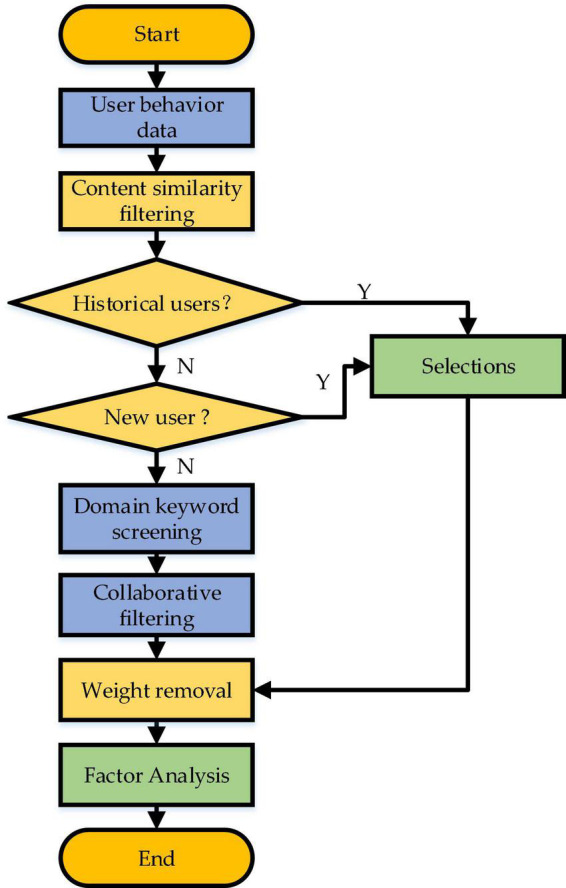
Recommended flowchart of hybrid IRS.

Then, this section presents a targeted user recommendation model to realize personalized recommendation by combining users’ different psychology for SRD needs to provide users with their interested SRD and literature efficiently. The recommendation system recommends relatively basic POI information for new users and the CBR-CF hybrid recommendation algorithm for historical users. Figure 2 specifies the implementation process. The algorithm trains the model by collecting user information. It obtains different users’ label information and makes personalized recommendations to users combined with resources.

### Construction of Scientific Research Data-Targeted Intelligent Recommendation System

#### Requirement Analysis of Intelligent Recommendation System

System requirements are mainly divided into Functional Requirement (FR) and Performance Requirement (PR). From the perspective of the design concept, the IRS needs to have functions, such as user behavior recording, role management, and real-time update. Specifically, the user behavior recording function traces the user’s browsing records and personal information to provide accurate recommendations according to these records; the role management function classifies users into two types: historical users and new users. Therefore, the IRS offers permission-varied services for role-based users. Lastly, the real-time update function tracks and analyzes the user’s instantaneous login information, browsing history, and browsing time to analyze the user’s POI, update their recommended content in real-time, and make optimal recommendations (Yuan and Wu, 2020).

**FIGURE 3:**
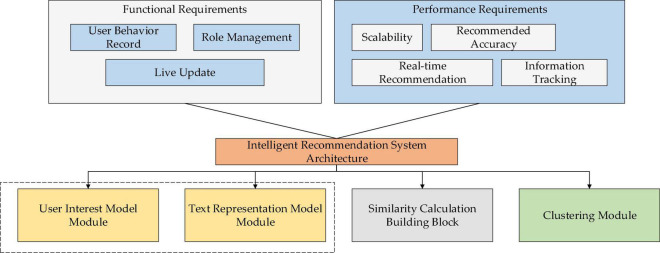
Framework of the proposed IRS.

The system performance requirements entail scalability, timeliness, recommendation accuracy, and the dynamicity of user information tracking (Victor, 2009). Recommendation services should be provided for users and updated in real-time. Meanwhile, the user’s dynamic POI should be timely mastered. The recommended information is supposed to meet users’ immediate needs. In particular, recommendation accuracy is a vital indicator of the performance of the IRS. Further, it is necessary to provide accurate and continuous information recommendation services until users’ actual needs are met. Tracking dynamics: personalized information recommendations should be based on quick and accurate acquisition of the users’ personalized information. First, it is necessary to conduct a real-time dynamic tracking analysis of the user’s behavior information to achieve accurate recommendations of the IRS (Shukla et al., 2015). The framework of the proposed IRS is shown in Figure 3.

The user POI module and text representation module affect each other, as explained in Figure 3. Text similarity analysis can help understand user similarity; users with similar interests will probably generate highly similar text representation. Therefore, the subsequent analysis will utilize the users’ text information.

#### Architecture Design of Intelligent Recommendation System

The proposed hybrid IRS obtains the user’s POI implicitly. According to the design requirements, four parts must be designed in the information IRS: users’ POI module, text representation module, similarity calculation, and the clustering module. The four-layer data structure is used for system function design, including the interface display layer, the recommendation analysis layer, the data processing layer, and the original data layer that stores the user’s behavior record information (Mabrouk et al., 2017).

Collaborative filtering recommendation uses domain-based ideas; that is, CF recommendation predicts target users’ POI through UB based on similar or the same interests; then, the degree of interest is calculated and used to determine specific recommendations. Thus, CF is widely used among scholars.

The original data layer mainly stores the user’s behavior information data (instant data and historical behavior information), background data, and the user’s identity information. The data processing layer is the core functional layer of the IRS. It mainly completes data cleaning, Word Segmentation (WS), word weights calculation, text analysis, and user feature vectors generation, and then completes the text and user clustering process. The recommendation analysis layer is used to determine the system’s recommendation algorithm and then use the optimal algorithm to recommend information to users. CBR-CF is implemented in the recommendation analysis layer. The interface display layer mainly provides an interface for Human-Computer Interaction (HCI).

The CBR-CF hybrid method makes up for both single CF and CBR, namely, the lack of personalization of CF and the lack of diversity of CBR. Thus, the hybrid algorithm can be used to overcome their respective deficiencies and obtain more accurate results.

### Process Design of System Module

The user, the user text input, similarity analysis, and clustering issues are the top priorities among the various IRS modules. The user POI and the input text representation strongly correlate; hence, the module designs are elaborated separately. Afterward, the user and text representation is completed for user similarity research. Here, the analysis is mainly based on the text representation input by the user, so the text-similarity is primarily analyzed in the subsequent analysis (Shen et al., 2019).

#### Design of User Curiosity Module Based on Psychology

Curiosity, a psychological term, is an essential factor that promotes the development of human cognition throughout their lifetime. Curiosity has always been considered the key motivation related to behaviors, such as exploration, investigation, and learning. Curiosity benefits an individual from both personal and social aspects. At the personal level, curiosity is related to personal growth, which is the individual’s passion for learning and knowledge, not diverted by benefits. Curiosity can strengthen interpersonal relationships by injecting energy and passion into social interaction at the social level. The close relationship between curiosity and human learning has inspired researchers to design curiosity computing models for ML systems to improve their learning ability to develop autonomous intelligent machines. Berlyne has conducted in-depth research based on curiosity-driven theory and put forward the curiosity arousal theory; that is, a user will only respond to stimuli that can arouse his curiosity. This theory is also called stimulus selection theory, which mainly discusses the mechanism of obvious stimulants introduction that humans will respond to. Berlyne argues that curiosity is the driving factor of curiosity arousal theory. Internally, curiosity drives people to choose specific stimulation. Externally, an individual’s stimulus-response can expose his curiosity.

**FIGURE 4:**
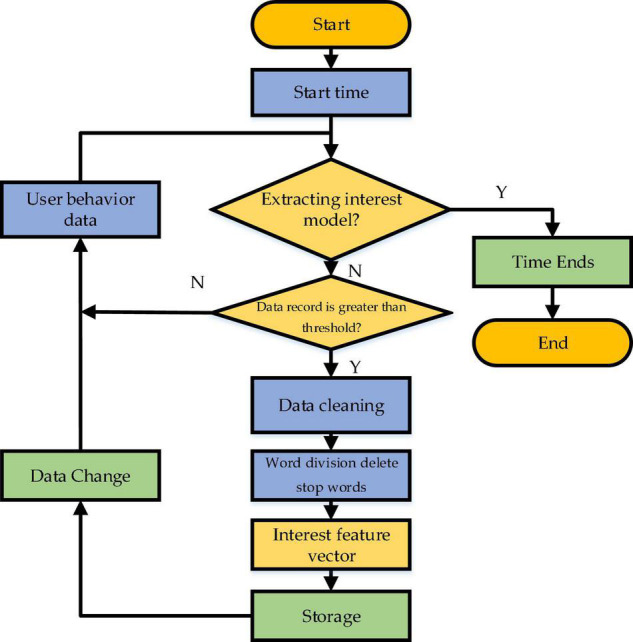
Flowchart of implementing user curiosity model based on Psychology.

Curiosity arousal theory holds that people’s curiosity characteristics can be predicted and estimated. In particular, the recommendation system can accurately understand users’ stimulation degree by simulating and predicting users’ curiosity and giving recommendations according to the stimulated curiosity degree. The user POI module is responsible for collecting user interest data and UB information and data-preprocessing, such as data cleaning, denoising, conversion, and filtering. This set of information should be updated timely. Meanwhile, the preprocessed data can be combined with relevant documents to remove the pause words. Then, the word weights are calculated, by which the words are sorted. This is a process of generating and sorting the feature vector of user POI, and data are updated by comparing the user’s log and the threshold. More precisely, the data are updated when the threshold is reached, and the data state is changed. Then, the feature vector is updated with a newly generated feature vector. Figure 4 draws the implementation flowchart of the user curiosity model based on Psychology. Apparently, the model explores the user’s curiosity by learning the user’s behavior data, judges the input behavior data, updates the data of the established curiosity model using the algorithm, and calculates the weight of the input vocabulary, thereby generating the feature vector of user POI.

#### Process Design of Text Representation Module

**FIGURE 5:**
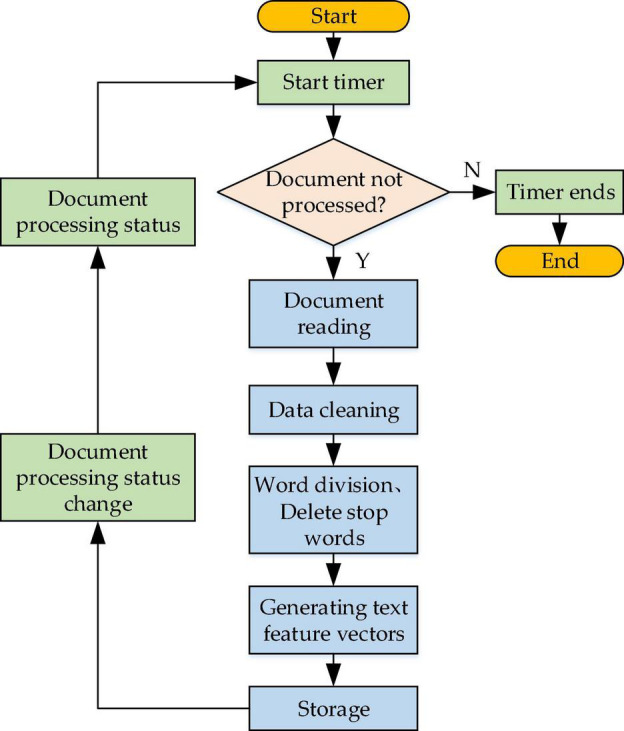
The flowchart of the text representation module.

A timer is designed into the text representation module to detect and update the text set periodically. Documents are supposed to be preprocessed through WS and stop words elimination first, and then the weight of words is calculated. Afterward, feature words are screened according to the weight ordering and spatial dimension information, and the final text feature vector should be generated for storage (text vectorization). The working process of the text representation module is shown in Figure 5.

#### Process Design of Similarity Calculation Module

The primary function of the similarity calculation module is to pick out users’ POI contents. Users’ interest feature vector can be combined with information prediction to avoid behavioral differences for users with common interests.

Meanwhile, an optimized similarity calculation often consumes less time and distinguishes different articles. Moreover, SRD formats are diverse, so the system should be designed to calculate different text formats. Finally, the cosine function is chosen to calculate the user similarity (Ranjbar and Alizadeh, 2017).

**FIGURE 6:**
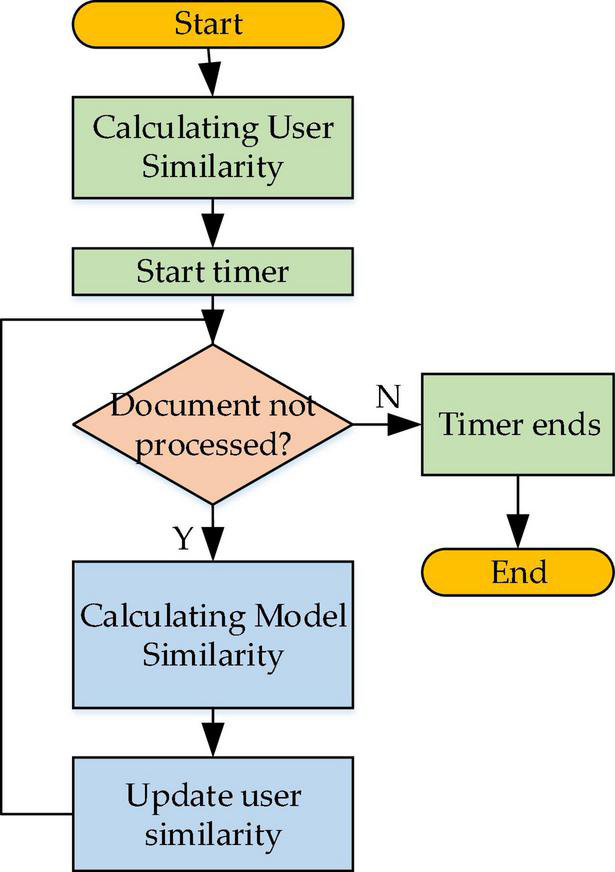
Working flowchart of the user similarity calculation module.

Similarity calculation is divided into user similarity calculation and text similarity calculation. User similarity calculation reads: first, the user similarity between sub-modules is calculated in the entire module. The real-time detection technology can calculate the updated content in real-time so that the similarity between users’ POI can be calculated timely. Figure 6 manifests the working process of the user similarity calculation module.

**FIGURE 7:**
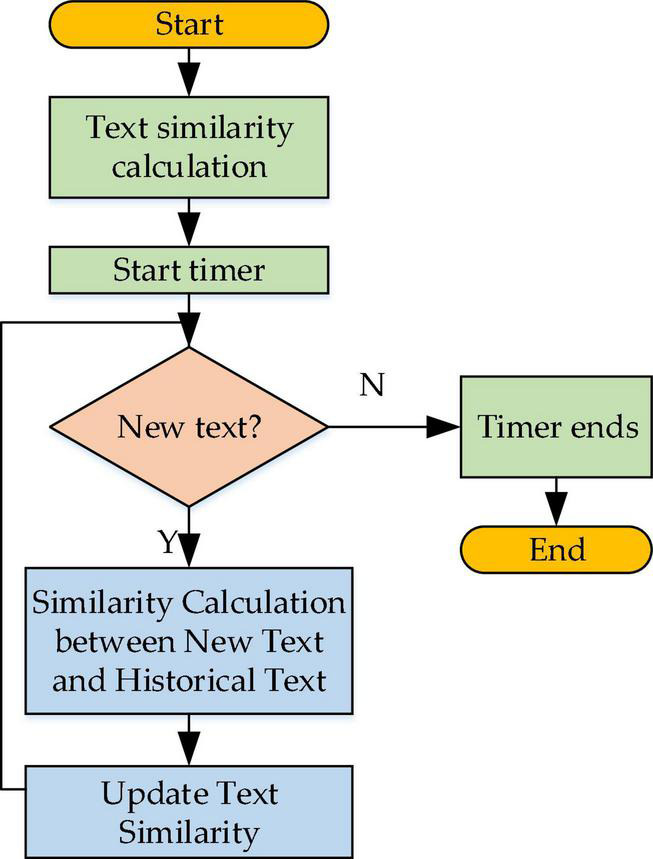
Working flowchart of the text-similarity calculation module.

Text similarity calculation process reads: first, the similarity between two texts is calculated, and then the text representation module is detected regularly for new text information. Next, the similarity between every piece of new text information and the existing text information is calculated and stored. Figure 7 outlines the working process of the text-similarity calculation module.

#### Process Design of Clustering Module

**FIGURE 8:**
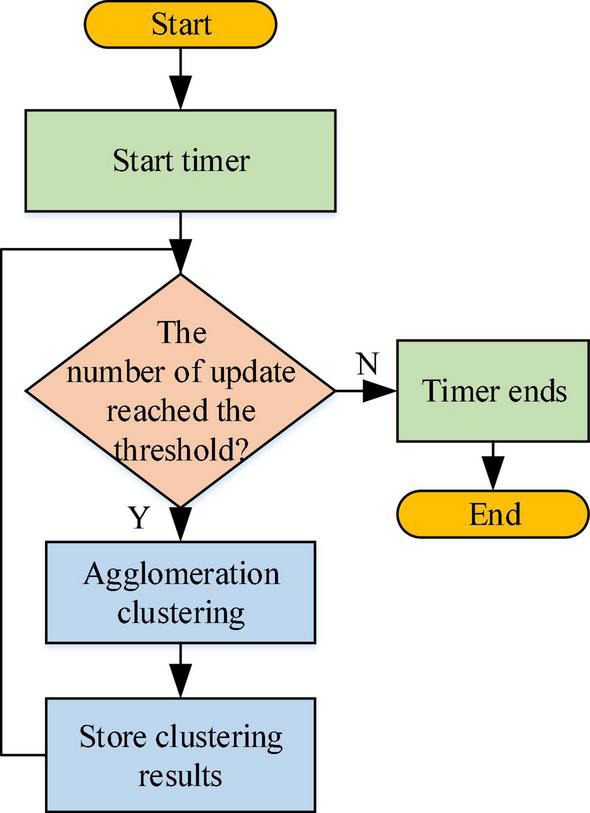
Working flowchart of clustering module.

The clustering algorithm based on hierarchical division is used to complete the recommendation of the user’s personalized information, and the optimized Chameleon algorithm is used for data clustering analysis. The algorithm can meet the timeliness of sampling, and the input of data has a small impact on the clustering effect of the algorithm. Specifically, the method of agglomerative hierarchical clustering is employed: first, the elements are clustered, the corresponding module degree is calculated, and then the clustering results are improved through iterative relocation (Zhang et al., 2018). Figure 8 shows the working process of the clustering module.

### Process Design of Content-Based Recommendation-Collaborative Filtering

**FIGURE 9:**
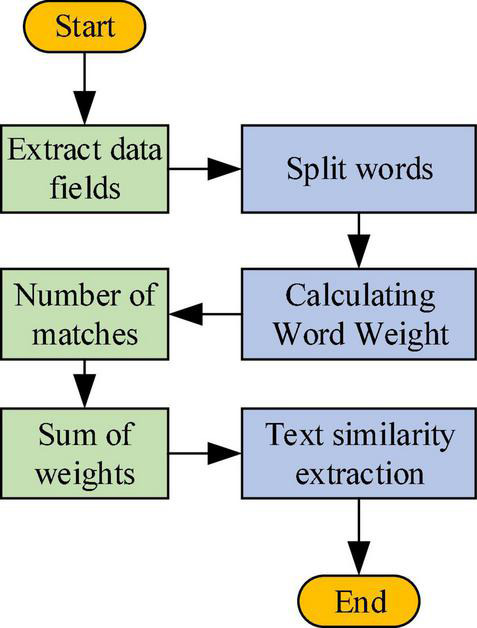
Calculation flowchart of similarity.

The design concept of the IRS of intelligence hybrid mainly includes two key points: word matching and weight design. When the algorithm is used for calculation and processing, the text data information is first obtained, the text data is initialized, and the key fields of the text, such as title, abstract, keywords, are extracted and processed, respectively. First, the text data are segmented, the stop words are deleted, and the weights of the fields are calculated separately. It is matched with the text information to be compared, the corresponding key-value pair is obtained, and the relative degree of the two is calculated. The calculation process of similarity is shown in Figure 9.

### System Indicator Analysis

The system is analyzed using accuracy, precision, and recall indicators. Accuracy is the ratio of correctly classified samples to the total number of samples in a given dataset. The precision determines the proportion of true samples to all samples determined to be true (Wang et al., 2013). Recall describes the proportion of positive cases judged to be true in the classification. *TP* (True Positive) indicates that a positive sample is correctly judged as a positive sample. *TN* (True Negative) means that a negative sample is correctly judged as a negative sample. *FP* (False Positive) signifies that a negative sample is incorrectly judged as a positive sample; *FN* (False Negative) indicates that a positive sample is incorrectly judged as a negative sample. The calculation of the above indicators reads:

(1) *Accuracy*: the correct classification rate of the classifier.


(1) $$A\,\text{c}\,c\,u\,r\,a\,y=\frac{T\,P+F\,N}{T\,P+F\,P+T\,N+F\,N}$$

(2) *Precision*: The total percentage of positive samples correctly classified by the classifier.


(2) $$P\,r\,e\,c\,i\,s\,i\,o\,n=\frac{T\,P}{T\,P+F\,P}$$

(3) *Recall*: the proportion of positive samples that are correctly predicted.


(3) $$R\,e\,c\,a\,l\,l=\frac{T\,P}{T\,P+F\,N}$$

The dataset mainly comes from existing SRD (Victor, 2009), 80% of which is used for algorithm model training, and 20% is used for algorithm model testing.

### Experimental Data Set and Parameter Setting

This section collects 2,000 articles in 10 research directions from the HowNet and Google academic websites, including material science, safety engineering, social science, environmental science, and computer science. Then, it divides the collected data set into the training set and test set with a ratio of 5:1. Afterward, different algorithms are used for the experiment. Subsequently, the optimal parameters for the experiment are obtained by continuously adjusting the parameters and comparing the experimental output results with the relevant research data. Meanwhile, the parameter setting of the hybrid algorithm is obtained by adjusting some main parameters of single algorithms.

## Analysis and Results

### Accuracy Analysis of Text Similarity Evaluation

**FIGURE 10:**
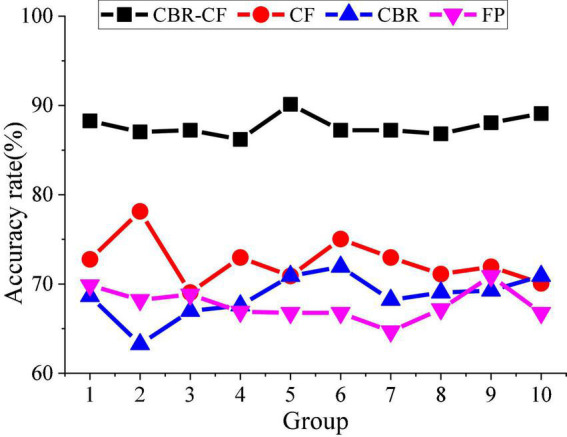
Analysis of the accuracy of the text-similarity calculation of the hybrid system.

Further, the recommendation performance of the proposed system is analyzed. First, the calculation accuracy of the text similarity is analyzed. All texts are divided into two parts: a training set and a test set, and the texts in the two sets are compared in pairs. Afterward, Figure 10 compares CBR-CF with CF, CBR, and FP algorithms, and the accuracy of the system similarity calculation method is obtained.

Figure 10 shows that the similarity calculation method of the hybrid system effectively reduces the dimension of discrete data and performs well on the weight calculation of fields, which improves the calculation accuracy and effectiveness and has high feasibility.

According to the similarity between adjacent data, data used in the corresponding literature are comprehensively considered; then, the two similarity data are merged to obtain the mixed data similarity finally. If the data are highly similar, the degree of data sparsity will be reduced effectively, thereby improving the system’s feasibility.

### Accuracy and Recall of Hybrid Intelligent Recommendation System

**FIGURE 11:**
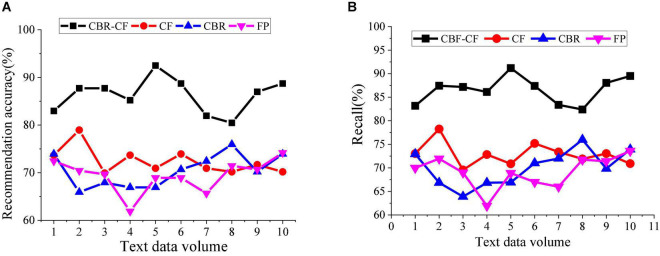
Comparison and analysis of the accuracy and recall of different recommendation systems. **(A)** Comparison analysis of accuracy rate of different recommendation systems. **(B)** Comparison analysis of recall of different recommendation systems.

This section analyzes the accuracy and recall of the hybrid IRS. The recommendation accuracy is obtained by the ratio of the browsing record of a single user to the recommended items, and the ratio also indicates the user’s interest in the recommended items. The recommended recall refers to the probability that the data the user wants to access is recommended. Figure 11 reveals the hybrid IRS’s recall and accuracy analysis results.

Figure 11 displays that under different recommendation methods, the accuracy of the proposed CBR-CF method is the highest. In particular, the accuracy of single-user system recommendations can reach 82–93%. The recall of all recommendation algorithms is 60–91%, while the recall of the CBR-CF algorithm is higher than that of a single algorithm, with the highest recall reaching 91%. The reason is that the CBR-CF algorithm combines ML technology and user Psychology, thereby improving the algorithm efficiency and accuracy. Therefore, the IRS based on the hybrid CBR-CF algorithm has better recommendation performance.

### Performance Analysis of Scientific Research Data-Targeted Intelligent Recommendation System

**FIGURE 12:**
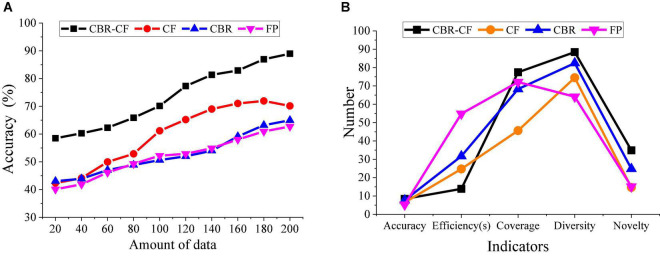
Comparison and analysis of the performance of different recommendation systems. **(A)** Analysis chart of recommendation accuracy of different recommendation systems. **(B)** Comprehensive comparison and analysis chart of the performance of different recommendation systems.

This section compares the performance of the proposed CBR-CF-based recommendation system and a single recommendation algorithm-based system using the evaluation accuracy for user information retrieval from the perspective of diversity, coverage, efficiency, and novelty of recommendation, as depicted in Figure 12.

Figure 12 conveys that under the same DV, the accuracy of the CBR-CF algorithm combined with ML technology and user Psychology is better than any single recommendation algorithm, and the accuracy of the CBR-Cf algorithm increases with DV. CBR-CF has higher recommendation accuracy and better recommendation effect and performance than a single recommendation algorithm. Figure 12A indicates that the highest accuracy of the designed CBR-CF algorithm is about 90%, while the highest accuracy of the CF algorithm is about 70%, the accuracy of the CBR algorithm is about 65%. The accuracy of the FP algorithm is about 60%. Thus, the CBR-CF algorithm has a better recommendation effect. Figure 12B suggests that CBR-CF performs best in efficiency, coverage, diversity, and novelty. Hence, CBR-CF has the excellent performance of a single recommendation algorithm and makes up for the shortcomings of a single recommendation algorithm, thereby improving the overall recommendation effect.

In other words, when analyzing user Psychology, the designed algorithm has higher recommendation accuracy than similar recommendation algorithms and can better deal with related problems. To sum up, the CBR-CF method can make a better recommendation, and the system’s accuracy and recall are different from the pure recommendation method. The accuracy of the proposed method has been improved by 8.3%. Therefore, this study provides a practical reference for developing SRD-targeted intelligent recommendation technology under the background of SD. In practical application, users can input their interested research content into the system, and the system will output the corresponding SRD, which can well meet user demands.

## Discussion

The CBR-CF algorithm combined with CBR and CF can solve the data sparsity and cold-start problem and expand the range of processable text types. At the same time, it improves the coverage of user recommendation information, increases the degree of automation, solves the system timeliness problem, and makes the system run more efficiently, which is consistent with the design concept of recommendation system in the literature (Zhao et al., 2017). Meanwhile, the CBR-CF algorithm is compared with the experimental results of similar algorithms. It is found that the designed algorithm has higher recommendation accuracy over similar algorithms and can better provide personalized recommendation services. Compared with the single recommendation method, the accuracy of the proposed method is only improved by 8.3%. The reason is that the recommendation by CF in the proposed hybrid method will be affected by specific data, which may affect the accuracy.

The research results can efficiently provide researchers in related fields with timely and relevant research materials. Overall, the actual recommendation performance of the proposed method is better than that of a single CF algorithm and CBR algorithm. Therefore, the hybrid algorithm has higher accuracy than the single algorithm, which is of great significance to the follow-up research of the recommendation system and provides more possibilities for SRD. In the future, researchers can use the SRD in the existing literature for system training and testing to give ideas for the design and implementation of IRS.

## Conclusion

The present work establishes an SD-oriented SRD-targeted IRS. The shortcomings are summarized following the literature review and analysis of the traditional single recommendation algorithms. Then, combined with ML technology and user Psychology, the CBR-CF algorithm is introduced. Afterward, a hybrid IRS based on the CBR-CF algorithm is proposed to improve the effectiveness of the CF method. Experiments show that under different recommendation methods, the accuracy of the proposed CBR-CF algorithm is the highest, and the highest recall is 91%. Additionally, under the same DV, the accuracy of CBR-CF is better than a single recommendation algorithm, and with the continuous increase of DV, the accuracy of CBR-CF is also improved. Finally, compared with other algorithms in efficiency, coverage, diversity, and novelty, it is found that the proposed CBR-CF algorithm shows good data advantages. Therefore, CBR-CF inherits the excellent performance of a single recommendation algorithm and makes up for their shortcomings, thereby improving the overall recommendation effect. The research results can provide a personalized SRD for researchers to effectively offer users with corresponding SRD recommendation approaches.

Still, there are some limitations: the research scheme cannot thoroughly analyze all semantic data, resulting in incomplete research results. Besides, only a few data set is used in the experiment. Therefore, the follow-up study will consider the influence of these factors, and the number of data sets should be increased. It is hoped that the scheme can provide some references for relevant researchers.

## Data Availability Statement

The raw data supporting the conclusions of this article will be made available by the authors, without undue reservation.

## Ethics Statement

The studies involving human participants were reviewed and approved by Zhejiang Wanli University Ethics Committee. The patients/participants provided their written informed consent to participate in this study. Written informed consent was obtained from the individual(s) for the publication of any potentially identifiable images or data included in this article.

## Author Contributions

All authors listed have made a substantial, direct, and intellectual contribution to the work, and approved it for publication.

## Conflict of Interest

The authors declare that the research was conducted in the absence of any commercial or financial relationships that could be construed as a potential conflict of interest.

## Publisher’s Note

All claims expressed in this article are solely those of the authors and do not necessarily represent those of their affiliated organizations, or those of the publisher, the editors and the reviewers. Any product that may be evaluated in this article, or claim that may be made by its manufacturer, is not guaranteed or endorsed by the publisher.
